# Computation-Assisted Identification of Bioactive Compounds in Botanical Extracts: A Case Study of Anti-Inflammatory Natural Products from Hops

**DOI:** 10.3390/antiox11071400

**Published:** 2022-07-19

**Authors:** Kevin S. Brown, Paige Jamieson, Wenbin Wu, Ashish Vaswani, Armando Alcazar Magana, Jaewoo Choi, Luce M. Mattio, Paul Ha-Yeon Cheong, Dylan Nelson, Patrick N. Reardon, Cristobal L. Miranda, Claudia S. Maier, Jan F. Stevens

**Affiliations:** 1 Department of Pharmaceutical Sciences, Oregon State University, 1601 SW Jefferson Way, Corvallis, OR 97331, USA; wbwu318@hotmail.com (W.W.); lucemattio@gmail.com (L.M.M.); dylan.nelson@oregonstate.edu (D.N.); fred.stevens@oregonstate.edu (J.F.S.); 2 School of Chemical, Biological, and Environmental Engineering, Oregon State University, 116 Johnson Hall, 105 SW 26th Street, Corvallis, OR 97331, USA; 3 Linus Pauling Institute, Oregon State University, 2900 SW Campus Way, Corvallis, OR 97331, USA; jamiesop@oregonstate.edu (P.J.); jaewoo.choi@oregonstate.edu (J.C.); cristobal.miranda@oregonstate.edu (C.L.M.); 4 Department of Chemistry, Oregon State University, 153 Gilbert Hall, Corvallis, OR 97331, USA; vaswania@oregonstate.edu (A.V.); alcazara@oregonstate.edu (A.A.M.); phyc.research@gmail.com (P.H.-Y.C.); patrick.reardon@oregonstate.edu (P.N.R.); claudia.maier@oregonstate.edu (C.S.M.)

**Keywords:** machine learning, Elastic Net, Random Forests, inflammation, bioassay, mass spectrometry, spectral network, *Humulus lupulus*, prenylated flavonoids, xanthohumol

## Abstract

The slow pace of discovery of bioactive natural products can be attributed to the difficulty in rapidly identifying them in complex mixtures such as plant extracts. To overcome these hurdles, we explored the utility of two machine learning techniques, i.e., Elastic Net and Random Forests, for identifying the individual anti-inflammatory principle(s) of an extract of the inflorescences of the hops (*Humulus lupulus*) containing hundreds of natural products. We fractionated a hop extract by column chromatography to obtain 40 impure fractions, determined their anti-inflammatory activity using a macrophage-based bioassay that measures inhibition of iNOS-mediated formation of nitric oxide, and characterized the chemical composition of the fractions by flow-injection HRAM mass spectrometry and LC-MS/MS. Among the top 10 predictors of bioactivity were prenylated flavonoids and humulones. The top Random Forests predictor of bioactivity, xanthohumol, was tested in pure form in the same bioassay to validate the predicted result (IC_50_ 7 µM). Other predictors of bioactivity were identified by spectral similarity with known hop natural products using the Global Natural Products Social Networking (GNPS) algorithm. Our machine learning approach demonstrated that individual bioactive natural products can be identified without the need for extensive and repetitive bioassay-guided fractionation of a plant extract.

## 1. Introduction

Humans receive daily exposure to natural products through consuming foods and botanical supplements. Many marketed botanical supplements are advertised to maintain or improve general health; consumers generally do not have access to evidence-based, unbiased information on the health benefits of botanical supplements. Even less is known about the bioactivity of their constituents. Unbiased testing of the usefulness and safety of botanical supplements has turned out to be more challenging than testing the efficacy and safety of single therapeutic agents. Despite recent advances in analytical chemistry and the availability of natural product databases, comprehensive chemical characterization of botanicals remains a time-consuming and labor-intensive endeavor. Assessing the health benefits of natural product-containing supplements is further complicated by their composition variability due to the environment's impact on secondary metabolism and the existence of subspecies and chemotypes. 

Unbiased pharmacological testing remains equally daunting because the many natural products in extracts usually have weak effects on multiple biological targets, which makes it difficult to assign bioactivity to single natural products or even a group of structurally related natural products. To complicate matters further, single natural products, or their metabolites, can exert pleiotropic effects by interacting with upstream pharmacological targets, such as nuclear receptors that regulate the transcription of genes in multiple signaling pathways. For instance, the principal prenylated flavonoid from hops (*Humulus lupulus*), xanthohumol, has been reported to exert anti-inflammatory effects, pro- and anti-oxidant effects, pro-apoptotic effects, as well as effects on targets relevant to cardiovascular and metabolic health [1] and cancer [2].

Hop prenylated flavonoids are produced by glandular trichomes, lupulin glands, and secreted together with humulones, lupulones, and essential oils in extracellular resin droplets, lupulin, which are visible as a yellow powder inside the bracteoles of the inflorescences of the female plant. The concentration of xanthohumol in lupulin ranges from 0.1% to 1%. Xanthohumol is detectable in beer at 0.5–4 mg/L [3]. Xanthohumol-containing hop extracts are commercially available and marketed as botanical supplements or used as an ingredient in nutraceutical formulations. In our studies of the anti-inflammatory activities of prenylated flavonoids from hops [4], we established structure–activity relationships using a cell culture model of inflammation. More recently, we established that xanthohumol attenuates diet-induced chronic inflammation in a mouse model of metabolic syndrome [5,6].

Identifying bioactive compounds from botanicals has traditionally used a labor-intensive and costly bioassay-guided fractionation approach. Multiple rounds of fractionation and testing are performed to obtain an isolated bioactive compound. After this expensive and time-consuming process, there is no guarantee of obtaining a novel compound. A more rapid method that could accelerate the discovery and dereplication of individual compounds from complex botanical mixtures would be a great asset to natural products research. For this reason, recent work has adopted a statistical/machine learning approach to the problem. Particular success has been obtained using a partial least squares regression model [7,8] to obtain information about bioactive compounds from chromatographic fractions [9,10]. In this case, the variable to predict is the bioactivity of each fraction, and the variables used for prediction are the MS intensities of each peak across all fractions.

Despite these successes, as we will show in this paper, data-dependent difficulties in this prediction problem require a more complex machine learning pipeline to achieve robust, reliable results over a wide range of input data. Our goals in this study are twofold. First, we present a general machine learning pipeline for bioactive component discovery; in this pipeline, virtually any kind of “learner” (like partial least squares) can be used as the core. We specifically test the Elastic Net [11] and Random Forest [12] models. Second, in our in vitro studies [4,13,14,15,16], animal [5,6,17,18,19], and human studies of xanthohumol [20] over the past 20 years, we always tested the hypothesis that xanthohumol or its structural analogs exerted bioactivity without paying attention to the other, more abundant natural product constituents of lupulin. In the present study, we used our pipeline to determine which lupulin constituents are responsible for the anti-inflammatory effects of a lupulin extract.

## 2. Materials and Methods

### 2.1. Reagents

LC–MS-grade methanol and water were purchased from EMD Millipore (Burlington, MA, USA). Formic acid ACS reagent was from Fisher Chemicals (Suwanee, GA, USA). DMEM was from Life Technologies (Grand Island, NY, USA). Penicillin, streptomycin and fetal bovine serum were from Invitrogen (Carlsbad, CA, USA). Xanthohumol (XN, purity > 99%) was provided by Hopsteiner Inc. (New York, NY, USA). Lipopolysaccharide from *Escherichia coli* (product no. L6529) was purchased from Sigma-Aldrich (St. Louis, MO, USA).

### 2.2. Plant Material

Dried hops (*Humulus lupulus* var. Taurus) were provided by Mr. Jeffrey Clawson, Manager of the Pilot Brewery in the Department of Food Science and Technology at Oregon State University.

### 2.3. Extraction and Extract Fractionation

Dried hops (10 g) were submerged in 200 mL acetone and stirred at 200 rpm for 3 h at room temperature. After the solids settled, the supernatant was collected and dried under mild nitrogen flow. The crude extract (lupulin, 2.1 g) was obtained and stored at −20 °C until further experiments were conducted. A portion of the crude extract (2 g) was dissolved in 10 mL of methanol. The solution was applied to a Sephadex LH-20 column (length 40 cm, diameter 5 cm), and the extract constituents eluted with 2.2 L of methanol at a flow rate of 4 mL/min. After collecting 750 mL of void volume, fractions of 20 mL were collected. The first 39 fractions were collected and dried under vacuum at room temperature. The remaining near-colorless fractions were combined, evaporated using a rotary evaporator, and termed fraction 40. The glass tubes were weighed before and after evaporation, so to obtain residue weights for each fraction.

### 2.4. Nitric Oxide (NO) Assay

**Figure 1:**
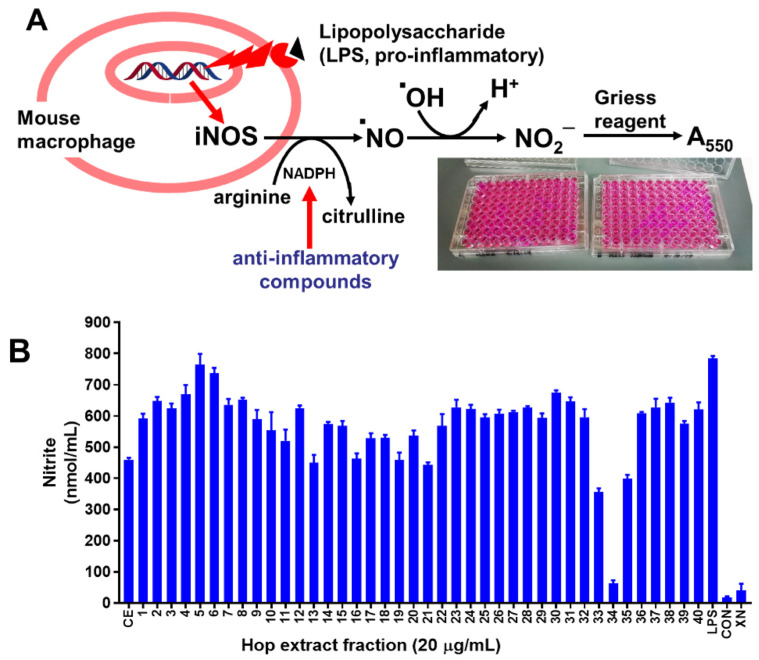
(**A**) Principle of the Griess assay to measure iNOS inhibitory activity; (**B**) iNOS Activity of the crude extract (CE), fractions 1–40, LPS (positive control), DMSO (negative control) and the hop constituent, xanthohumol (XN, 20 µM). Each fraction was measured in triplicate.

The bioactivity of the individual Sephadex fractions and the original crude extract was evaluated by determining the ability of these test materials to inhibit the formation of NO in RAW 264.7 cells activated by LPS. (See Figure 1 for a schematic of this assay.) NO secreted into the culture medium was determined as nitrite by the Griess reagent. RAW 264.7 murine macrophage cells obtained from the American Type Culture Collection (Manassas, VA, USA) were first cultured in 75 cm^2^ culture flasks containing DMEM supplemented with 2 mM glutamine, antibiotics (100 U/mL penicillin and 100 µg/mL streptomycin), and 10% heat-inactivated fetal bovine serum. When the cells reached 80% confluency, the cells were trypsinized and seeded in 96-well plates (0.2 mL/well) at a density of 1.5 × 10^5^ cells/mL. After 24 h, the cells were co-treated with LPS (1 µg/mL) and the test materials at a final concentration of 20 µg/mL. The test materials (crude extract and fraction residues 1–40) were first dissolved in DMSO before adding to the wells of the culture plate. Vehicle control cells were treated with 0.1% DMSO alone. Untreated cells (no LPS, no test material) grown in the complete DMEM culture medium served as negative controls. XN (99% purity) dissolved in DMSO was added to positive control cells at a concentration of 10 µM. Culture media from the control and treated cells were collected after 24 h of incubation and were frozen at −80 °C before analysis. For the determination of NO (as nitrite), 100-μL aliquots of the thawed culture media were mixed with an equal volume of Griess reagent (1:1 mixture (*v*/*v*) of 1% sulfanilamide and 0.1% naphthylethylenediamine dihydrochloride in 5% H_3_PO_4_) on a 96-well plate. The samples were incubated for 10 min at room temperature and then the absorbance was read at 550 nm using a SpectraMax 190 (Molecular Devices, Sunnyvale, CA, USA) plate reader. Nitrite was quantified using a standard curve generated by reacting nitrite in increasing concentrations with the Griess reagent on the same plate containing the test samples.

### 2.5. Mass Spectroscopic Analysis of the Crude Extract and Extract Fractions

Crude extract and the 40 fractions were reconstituted in methanol at a concentration of 200 ng/mL sample and spiked with ^13^C_3_-xanthohumol (internal standard) at a concentration of 40 ng/mL. ^13^C_3_-Xanthohumol was synthesized in previous work [21]. Samples were centrifuged at 13,000 rpm for 4 min and analyzed by loop-injection (LI) combined with electrospray ionization and high-resolution accurate mass spectrometry (LI-ESI-TOF). The analysis was conducted using a Shimadzu Nexera UHPLC system connected to an AB SCIEX TripleTOF^®^ 5600 (Concord, ON, Canada) mass spectrometer. For negative ion mode acquisition, the following parameter settings were used: spray voltage −4200 V; source temperature 550 °C, and a duty cycle time of 150 ms was used. For ESI+ acquisitions, the instrument settings were the same as those used in the negative ion mode, except that the spray voltage was set to 4500 V. The mass spectrometer was equipped with a calibrant delivery system. One blank sample was added between every three samples. Mass calibration was automatically performed every two hours. For loop-injection sample introduction, a 1 mm C8 guard column was used instead of an analytical LC column. The flow rate was 0.2 mL/min, and the injection volume was 5 µL. The guard column was eluted using a gradient with solvent A—water and solvent B—acetonitrile, both acidified with 0.1% (*v*/*v*) formic acid. The elution gradient was as follows: 0–2 min, held at 2% B; increase from 2% to 100% B over the next 0.1 min; held at 100% B from 2.1 to 3.9 min; then, decrease to 2% B in 0.1 min and run for another min to equilibrate. Therefore, the total run time was 5 min/sample and the total analysis time. MarkerView 1.3.1 software was employed to process and normalize the data. The data were subjected to a de-isotoping/declustering algorithm (Progenesis QI v3.0, Nonlinear Dynamics, Waters Corporation, Milford, MA, USA) to remove isotopologues and adducts of the same molecular species.

To obtain MS/MS fragmentation spectra for all constituents present in the crude extract, the crude extract was subjected to LC-QTOF-MS/MS analysis. Chromatographic separation was conducted using a Shimadzu Nexera UPLC system equipped with an Inertsil Phenyl-3 column (150 × 4.6 mm, 5 µm). Mobile phase A was water containing 0.1% formic acid, and mobile phase B was methanol containing 0.1% formic acid. The gradient started with 5% B and was held for 1 min, followed by a 10 min linear gradient from 5% to 30%. The gradient was then stepped to 100% B at 23 min and held for 12 min, and finally, stepped back to 5% B to equilibrate the column. The flow rate was 0.4 mL/min, and the column temperature was maintained at 45 °C. The UPLC system was connected to an AB Sciex Triple TOF 5600 mass spectrometer equipped with a TurboSpray electrospray ionization source operated in the negative ionization mode. The instrument was operated in the information-dependent acquisition (IDA) mode using a collision energy setting of 40 V. Peak integration parameters were as follows: LC minimum peak width, 5 s; noise threshold was 1000 for negative, and 3000 for positive ion mode. The *m*/*z* peak width was 40 ppm. Other conditions were the same as in the LI-QTOF experiment. Extract constituents were characterized by retention time, accurate mass, isotopic pattern, and MS/MS spectra and identified by LC-MS/MS comparison with 500 authentic standards (Natural Products Library, Enzo Life Sciences, Farmingdale, NY, USA).

### 2.6. Elastic Net Analysis

For each Elastic Net model in the model ensemble (described in model Results), we transform the input MS intensity as log(1 + *I*) and the response variable (bioactivity) as log(y). Parameter estimation for the elastic net was via the R package caret [22], which uses internal cross-validation (splitting of the training data) to find the parameters which determine the overall amount of regularization in the model (lambda) and the relative mixture of ridge and lasso terms (alpha). Regression coefficients (which yield model importances) are then extracted from the best cross-validation model. We note that this cross-validation step is in addition to the ensemble train/test splits—the test data is never shown to the model during parameter estimation. We then calculate importances $I_{j}^{\left(i\right)}$ and weights $w^{\left(i\right)}$ as described in the Results section.

### 2.7. Random Forests Analysis

For the random forest models, we logarithmically transform the predictors and response variables as we do in the Elastic Net above. We use a two-step training process for each Random Forest that uses the R packages randomForest [23] and caret [22]. We first use the out-of-bag error to determine the number of trees in the forest. Once we have chosen the number of trees, we use leave-one-out cross-validation (again, only the training data for that particular model in the ensemble) to determine the number of randomly sampled features (peaks) to use at each split in the forest. We then use this final optimized model to calculate permutation importances $I_{j}^{\left(i\right)}$ and weights $w^{\left(i\right)}$, as described in the Results section.

### 2.8. Spectral Network Analysis

High-resolution MS/MS data were converted into mzXML format using MS convert V3.0 loaded into MZmine V2.37 [24,25], which produces a feature table in a CSV file that aligns the spectral data in a MGF file. We used the latter file as input for the Global Natural Products Social (GNPS) molecular networking algorithm [26]. The resulting molecular networks were visualized in Cytoscape V3.6.1.

### 2.9. Bioassay Validation of Xanthohumol

The concentration-response range of XN (99% purity) bioactivity was determined by evaluating the ability of XN to inhibit the formation of NO in RAW 264.7 cells activated by LPS. NO production was determined as nitrite by the Griess reagent. RAW 264.7 cells were cultured in 75 cm^2^ culture flasks containing DMEM supplemented with antibiotics (100 U/mL penicillin and 100 µg/mL streptomycin) and 10% heat-activated fetal bovine serum. At 80% confluency, the cells were trypsinized and seeded in 96-well plates (0.2 mL/well) at a density of 1.5 × 10^5^ cells/mL. After 24 h, the cells were treated with LPS (1 µg/mL) and XN through a 2-fold serial dilution at a final concentration range of 60 µM to 0.46 µM. XN was first dissolved in DMSO before being added to each well, and each well was normalized to receive the same amount of DMSO (0.1%) by microfluidics HP D300e Digital Dispenser (Palo Alto, CA, USA). Vehicle control cells were treated with 0.1% DMSO alone. Untreated cells (no LPS, no XN) grown in the complete DMEM culture medium served as negative controls. After 24 h incubation, culture media from control and treated cells were collected and stored at −80 °C before analysis. NO determination (as nitrite) was assessed by Griess reagent. Briefly, 100 µL of room temperature culture media were mixed with an equal volume of Griess reagent (1:1 mixture (*v*/*v*) of 1% sulfanilamide and 0.1% naphthylethylenediamine dihydrochloride in 5% phosphoric acid) on a 96-well plate. The samples were incubated in low light for 10 min at room temperature, and the absorbance was read at 550 nm using a Spectramax 190 (Molecular Devices, Sunnyvale, CA, USA) plate reader. Nitrite concentration was determined using a standard curve generated by reacting Griess reagent with increasing concentrations of nitrite.

### 2.10. Quantitative Analysis of Inhibitory Fractions

For quantitation of XN in extract fractions 30 through 38 that showed iNOS inhibition, liquid chromatographic separation and mass spectrometric detection were performed using a Shimadzu 20AD system (Shimadzu, Columbia, MD, USA) coupled with electrospray ionization (ESI), hybrid triple quadrupole linear ion trap mass spectrometer (AB SCIEX, Framingham, MA, USA). Fractions were reconstituted in 50% methanol at a concentration of 9 µg/mL and spiked with ^13^C_3_-Xanthohumol as an internal standard at a concentration of 100 ng/mL. Samples were measured in triplicate with blank samples inserted between every three samples. The chromatographic separation was performed on an Agilent C8 reversed-phase column (2.1 × 50 mm, 3.5 µm), with an injection volume of 3 µL and a total flow rate of 0.4 mL min^−1^. A gradient with two mobile phases (A: 0.1% formic acid in the water, B: 0.1% formic acid in acetonitrile *v*/*v*) was as follows: solvent B increased from 30% to 60% from 0–1.5 min, was held at 60% from 1.5–2.5 min, increased to 100% until 3.0 min, was held at 100% from 3.0–3.8 min, decreased to 30% at 3.9 min, and the column was equilibrated until 6.0 min. The column effluent was introduced into the ESI source operated in negative ion mode with the following parameter settings used: spray voltage −4000 V; temperature 450 °C; CUR 35 L min^−1^; GS1 50 L min^−1^; GS2 45 L min^−1^, total run time per sample 6.0 min. Selected reaction monitoring (SRM) transitions for quantification were: *m*/*z* 353→119, *m*/*z* 353→233 for XN and IXN and 356→120, 356→234 for ^13^C_3_-XN (internal standard). Quantitation analysis was performed using AB Sciex MultiQuant software. External ten-point calibration curves were prepared for XN and IXN (R values > 0.995). XN and IXN were identified by matching retention time, isotopic pattern, the exact mass of the [M-H]^−^ ion and fragmentation pattern with that of known standards.

### 2.11. Analysis of Late Fractions

To determine what compound(s) were responsible for the decrease in XN-mediated inhibition in fractions 35–38, we computed the difference between predicted iNOS inhibition in those fractions, using the XN dose-response, and the observed amount of iNOS inhibition. We then fit a series of one-variable (plus intercept) regression models to the difference data in fractions 35–38, using each feature/peak in the MS data. Both the features (peak intensities) and the response variable were log-transformed as above. We judged model quality by residual error and model *p*-value, which in this case is just the *p*-value of the single fitted coefficient in the model.

## 3. Results

### 3.1. Bioactivity Testing of Fractions and Model Input

#### 3.1.1. Bioactivity Testing of Fractions

We fractionated an acetone extract of dried hop cones on a Sephadex LH-20 column using methanol as the eluant and collected 40 fractions. We tested the crude extract as well as all fractions for iNOS inhibitory activity by determining nitric oxide (NO) release from lipopolysaccharide (LPS)-treated RAW 264.7 cells and measured NO as nitrite using the Griess assay [27]. Figure 1 shows the principle and the results of the assay. Stimulation of the cells with LPS resulted in a nitrite concentration of 785 nmol/mL, whereas the negative control, DMSO, yielded a nitrite concentration of about 17.9 nmol/mL. The crude extract and the extract fractions yielded nitrate concentration in the range 63.6 nmol/mL (fraction 34) to 764 nmol/mL (fraction 5), showing that various fractions inhibited iNOS more or less than the crude fraction, which produced 460 nmol/mL nitrite. In addition, pure xanthohumol was tested at a concentration of 20 µM (=7.1 µg/mL) (Figure 1).

We determined the concentration-response curve of pure xanthohumol for iNOS inhibitory activity by measuring nitric oxide (NO) release as nitrite using the Griess assay in lipopolysaccharide (LPS)-treated RAW 264.7 cells. Stimulation of the cells with LPS alone resulted in 785 nmol/mL nitrite concentration, while negative controls, with DMSO, resulted in 24.9 nmol/mL nitrite concentration (Figure 1).

#### 3.1.2. Input Data to the Model

As input to our computational pipeline, all fractions were subjected to loop injection (LI)-HRMS. Raw MS data of hops fractions were processed by MarkerView 1.3.1 software to remove isotopic peaks and adducts. We obtained 236 peaks (64 for ESI− and 172 for ESI+) in the final list, of which the negative ion features were used for developing the predictive models. These peaks form the predictors and the nitrite concentration of the “bioactivity” variable to predict the computational methods we describe subsequently. Note for what follows that neither peak cleanup nor our subsequent reproducibility filtering changed the tag numbers, making it possible to have tag numbers larger than 64 in the ESI− data.

### 3.2. Computational Discovery Pipeline

#### 3.2.1. Challenges

**Figure 2:**
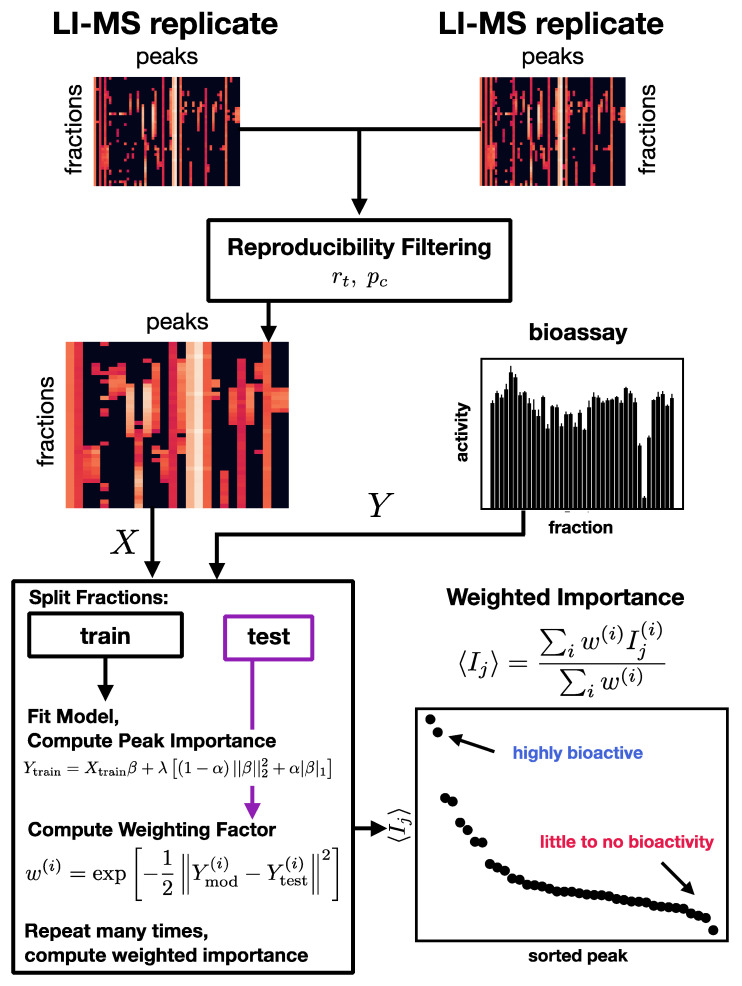
Computational pipeline developed for this study. Multiple LI–MS replicates are used to filter out nonreproducible MS peaks. The filtered MS data is then combined with the bioassay results and an ensemble of models created. Variable importance—the degree to which each individual MS peak contributes to the bioactivity—is computed from the model ensemble and used to identify bioactive compounds.

In developing a machine learning pipeline to associate molecular ions with bioactivity in the lupulin extract, we encountered three challenges in the data that required special attention. We believe these features are not specific to the lupulin extract in this study but will be shared broadly across other botanical extracts and complex mixtures. In what follows, we refer to the fractions as “samples” and the intensity of each ion across all the fractions as “features.” We first list these challenges and then discuss our solution to each, which defines our computational pipeline (shown in Figure 2).

1. In order to keep the full-scan QTOF-MS data in the linear range (so peak intensity is linearly proportional to concentration), the samples submitted to QTOF-MS analysis must be relatively dilute. This is essential for the modeling but can result in poor replicate-to-replicate reproducibility for some peaks that can lead to suboptimal model results.
2. We have a limited number of samples (~40) when compared to MS peaks (tens to thousands); this makes predicting the bioactivity (and identifying important features) from the MS data a “wide” statistical problem [28,29] that requires sophisticated regression models in order to be successful.
3. The standard method for parameter estimation in machine learning is cross-validation [30]. The data is divided into training and testing data, and the model parameters are then determined by fitting to only the training data. Then, the model goodness-of-fit is assessed relative to the test data, which the model has never seen. While this is generally a sound strategy, it relies on the assumption that the testing and training data come from the same underlying distribution—with thousands of samples or more that are split into training and test sets, one can be relatively certain this is the case. However, with a small number of samples—especially the lupulin extract in which bioactivity is not broadly shared across all fractions (see Figure 1B)—this can be easily violated and lead to unstable model estimates, even if cross-validation is used.

#### 3.2.2. Reproducibility Filtering

We wanted to use similarity between multiple MS replicates to construct more robust sets of features by averaging to both reduce noise and remove peaks that replicate poorly. An obvious solution to this problem would seem to be as follows. Using technical duplicate analyses from the same fractions, compute replicate–replicate correlations for each feature (interclass correlation coefficients [31,32] if you have more than two replicates) and exclude features below a certain correlation threshold. For the features that pass the threshold, use the average across the multiple replicates as the feature vector. In what follows, we discuss the difficulties with this naïve approach and our modifications to the procedure specifically for two replicates appropriate for the lupulin extract data.

**Figure 3:**
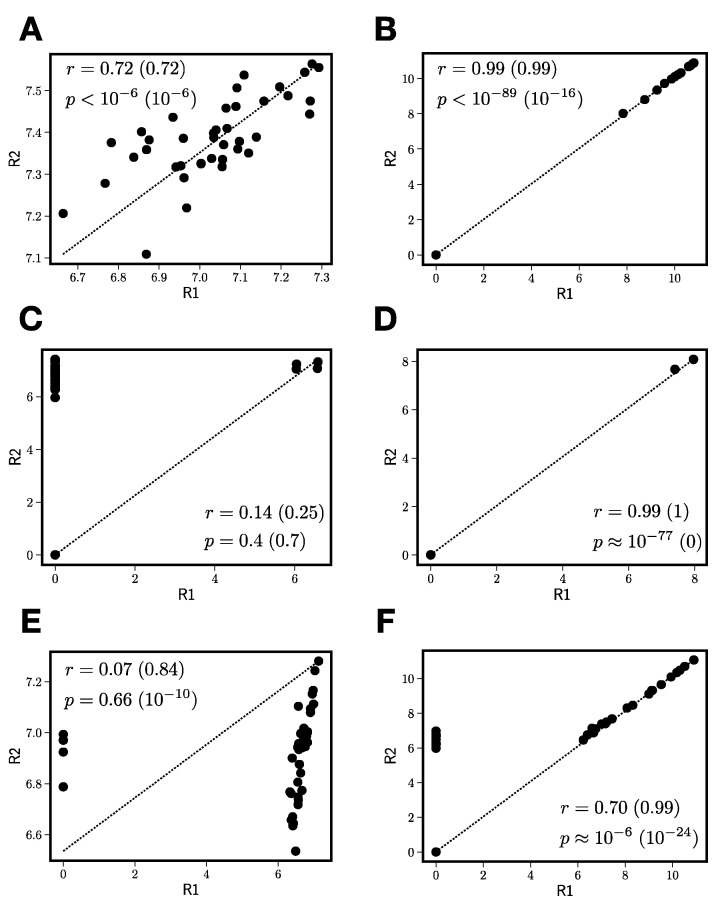
Log of one plus peak intensity, plotted for six different peaks (**A**–**F**) with one replicate (R1) plotted against another (R2). (**A**–**C**) All denote non-problematic peaks for “naïve” reproducibility filtering. (**D**) Denotes a peak with artifactually high correlation, and (**E**,**F**) show peaks that would be differentially selected based on standard versus zero-censored cross-replicate correlation calculations. Diagonal lines indicate perfect correlation.

Figure 3 explains why the naïve approach to reproducibility filtering performs poorly. The difficulty boils down to problematic peaks that have zero intensity in some replicates and nonzero intensity in others. In Figure 3, we show log(1+I), where I is peak intensity for six different *m*/*z* peaks in the negative mode MS lupulin extract data, with values in one replicate (R1) plotted against the second replicate (R2). The Pearson correlation values (r) and associated p-values (p) are shown in the insets to the panels, along with “zero-censored” correlations and p-values in parentheses. Zero-censored correlations are computed for only the portion of the data in which both replicates have nonzero peak intensity; the reason for showing both the censored and uncensored correlations will be clear as we discuss the details in Figure 3.

Figure 3A–C show unproblematic peaks. Figure 3A,B are highly reproducible. Figure 3A has a nonzero intensity in all fractions in both replicates. Figure 3B has zero intensity in some fractions in both replicates, but the nonzero intensities are almost perfectly correlated, and the comparison between the zero-censored and normal correlations shows no differences. These peaks would be included as features for further analysis using any reasonable correlation cutoff. Figure 3C is differentially zero in many fractions (note the vertical band of zero intensity in sample R1) and shows poor reproducibility.

Figure 3D shows a peak that appears to be extremely reproducible, but this turns out to be artifactual. This is because the peak in Figure 3D has a nonzero intensity in only two of 40 fractions; the censored correlation is perfect, but two data points are always (trivially) perfectly correlated, since a line can be drawn through the two points that pass through them perfectly. Peak Figure 3D indicates the reproducibility is uninformative or cannot even be defined for any peak with less than three jointly nonzero intensities.

Figure 3E,F are the most interesting of the set, yielding a rationale for using zero-censored correlations when calculating reproducibility. Figure 3E has a charge-to-mass ratio corresponding to xanthohumol. However, its uncensored correlation and p-value are quite poor. This is because, in four fractions, xanthohumol has a nonzero intensity in one replicate and zero in the other. This would cause xanthohumol to be excluded from further analysis based on this lack of reproducibility. However, once we compute the zero-censored correlation, we see xanthohumol has a very high, extremely significant cross-replicate correlation. Unlike xanthohumol, the peak in Figure 3F would generally be selected for inclusion under either standard or zero-censored correlation, but naïvely averaging the two replicates would reduce the quality of the resulting peak in the fractions that are differentially zero.

Figure 3 and the attending discussion lead us to the following algorithm for reproducibility filtering of the MS data before statistical learning:

For each peak:

1. Compute the number of jointly nonzero elements n_G_.
2. If n_G_ is less than 3, exclude this peak from further processing.
3. For peaks that pass the nonzero element criterion, compute zero-censored correlations r and associated p-values between $\log\left(1+I_{1}\right)$ and $\log\left(1+I_{2}\right)$.
4. Correct zero-censored p-values for multiple testing using the Benjamini–Hochberg false discovery rate [32].
5. If $p_{c}$< 0.05 and r > $r_{t}$, construct the replicate average peak by averaging only over the jointly nonzero values in the two replicates.

For the correlation threshold for inclusion $r_{t}$, we have experimented with both a “soft” threshold of 0.5 (corresponding to 25% of variance explained in one replicate by the other) and a “hard” threshold of 0.71 (corresponding to 50% variance explained). This process is shown schematically in Figure 2, in which two replicates are combined into a single set of peak intensities.

#### 3.2.3. Wide Problems

A second challenge in the bioactive NP discovery problem is the ratio of features to samples. Typically, we will have 20–40 chromatographic fractions, but depending on the details of the peak picking algorithm, tens to hundreds of features (measured charge-to-mass peaks). If peak picking is performed extremely conservatively or not at all, the number of features can be in the thousands. This kind of problem—in which there are far more explanatory variables than available samples—is known in the statistics literature as a “wide” problem [28]. Wide problems generally require some sort of regularization to help them sift through the excess explanatory variables. Regularization trades bias in the estimated model coefficients for a reduction in variance in their estimates.

In our approach, we consider two models: the Elastic Net [11] and Random Forests [12]. We choose the Elastic Net because it is a linear, regularized model, very suitable for wide problems. The Elastic Net combines two forms of regularization: L2, as used in Ridge Regression [33], and L1, as used in the Lasso [34]. The lasso is highly effective in shrinking regression coefficients to zero, and the ridge is effective at equalizing regression coefficients across sets of correlated features. The Elastic Net is a linear model, which is advantageous because nonlinear models (like neural networks) typically need large amounts of data for training, and because assessing the role of an individual feature (mass peak) in such models can be difficult [35]. We prefer the Elastic Net to Partial Least Squares Regression (PLSR [8]) because, in PLSR, the transformation of the original feature space into linear combinations of features makes credit/blame assignment in the fitted model less straightforward than the Elastic Net. PLSR has been previously used for bioactive natural product discovery [9,10], but not in the way we do in this study. We also consider Random Forests, which can partition the data space more finely than linear regression models, making them potentially more powerful. We emphasize that one could use virtually any statistical learning model as the basis for the model ensemble, which will become clearer when we describe our ensemble approach below.

#### 3.2.4. Variable Importance

Regardless of the particular statistical learning model chosen, our goal is not simply to fit the bioactivity data but to map this back to the individual mass peaks in the MS data, meaning, which compounds are actually responsible for making good predictions about bioactivity? This means we want to assess variable importance. The importance of a variable in a learning model depends on the model structure; for example, neural networks have very different definitions of variable importance [36] than do Partial Least Squares [37]. For the Elastic Net, we define a variable’s importance as follows:

$$I_{j}=\left|sgn\left(\beta_{j}\right)\right|,$$

where $\beta_{j}$ is the estimated regression coefficient for variable j. This measure simply assigns a score of unity to all peaks that appear in the fitted model with nonzero regression coefficients. These importance values are used to construct the ensemble average importance (see below), which we use to rank mass peaks by their bioactivity.

Random Forests have different importance measures; we use permutation importance [12]. The importance of a variable j in this definition is how much worse the model prediction becomes when the fitted model is evaluated with variable j scrambled (randomly permuted). This scrambling disrupts the variable’s relationship with the outcome (the bioactivity). If the scrambled variable is simply fitting noise, prediction quality will not drop much, and the variable is unimportant. If, on the other hand, that variable is necessary for fitting the data, the prediction quality will drop markedly, and the variable will be deemed important. Unlike the binary definition of importance for the Elastic Net above, permutation importance yields a continuous value for each variable.

#### 3.2.5. Model Ensembles

The final challenge is the relatively limited number of fractions available for analysis. As mentioned previously, meaningful cross-validation rests on the fact that samples used for model training are statistically similar to those used for model testing/validation. With hundreds of thousands of samples, this is usually not a concern; however, with the 40 fractions we have in this study, it is more worrisome. Exacerbating this problem is that, in our case, bioactivity is confined to a handful of fractions and not broadly shared across the fractions (see Figure 1B). Because of this, random splits of the data into training and testing fractions can produce very unstable model estimates from split to split, depending upon which fractions are included in the two sets. We see this behavior in single model runs of both the Elastic Net and Random Forest models, as well as other models we tried (data not shown). The important features/peaks differed dramatically from run to run. Another ever-present concern, particularly with powerful models and limited data, is overfitting, in which one can produce excellent fits of the data used to calibrate the model, but predictions of novel or unseen data are poor.

We adopted an ensemble approach [38] geared specifically toward prediction to address these issues. This process is schematically illustrated in the lower left box in Figure 2. We split the data randomly into training (2/3 of the data) and test (1/3) sets, $\left(X_{train}^{\left(i\right)},Y_{train}^{\left(i\right)}\right)$ and $\left(X_{test}^{\left(i\right)},Y_{test}^{\left(i\right)}\right),$ respectively. We use the training data to estimate the model at hand, which is performed using an internal cross-validation approach employing only the training data. We use standard methods for parameter fitting in both the Elastic Net and Random Forests (see Methods). We then use the test data to construct a weight for each model in the ensemble, as follows:

$$w^{\left(i\right)}=\exp\left(-\frac{1}{2}\|Y_{mod}^{\left(i\right)}-Y_{test}^{\left(i\right)}\|{}^{2}\right).$$

The term inside the exponential is half the squared error of the model when comparing its predictions $Y_{mod}^{\left(i\right)}$ of the *test* data (bioactivity of the test fractions) to the experimental results from the test fractions $Y_{test}$. Overall, this term is like a model likelihood for the *test* data, assuming Gaussian distributed independent errors. Notice the training data is not used here; to avoid rewarding models that overfit, we assess each model’s quality on the test data only. We repeat this process with a new random 2/3–1/3 split of the data hundreds or thousands of times.

For every data split, we also compute importance $I_{j}^{\left(i\right)}$ for each feature (peak) j from each of the hundreds or thousands of models (index i) as described above. We combine the results of all the splits using the weighting factors as follows:

$$\langle I_{j}\rangle =\frac{\sum_{i=1}^{N}w^{\left(i\right)}I_{j}^{\left(i\right)}}{\sum_{i=1}^{N}w^{\left(i\right)}}.$$

This calculation ensures that the ensemble of important peaks constitute those *that are important in predicting bioactivity values repeatedly in models which are themselves highly predictive overall.* A typical weighted importance graph is shown in the lower right of Figure 2, which shows the values of $\left\langle I_{j}\right\rangle$ for each peak, sorted from most important (left) to least important (right). While there is no clear cut-off from important to unimportant, we typically see the same steep drop in importance after a few very important peaks, such that after approximately ten peaks, everything else is irrelevant.

For a mixture/extract in which bioactivity is more broadly shared across the fractions, fewer models are needed to stabilize the average, and the results from our pipeline will trend more towards analysis from a single model, assuming similar reproducibility filtering had been done in both cases. Our methods would thus agree with a simpler computational pipeline for “easy” cases but also allow us to achieve robust results in “hard” cases that simpler pipelines would not be able to produce.

### 3.3. Model Results and Validation

#### 3.3.1. Model Results

**Figure 4:**
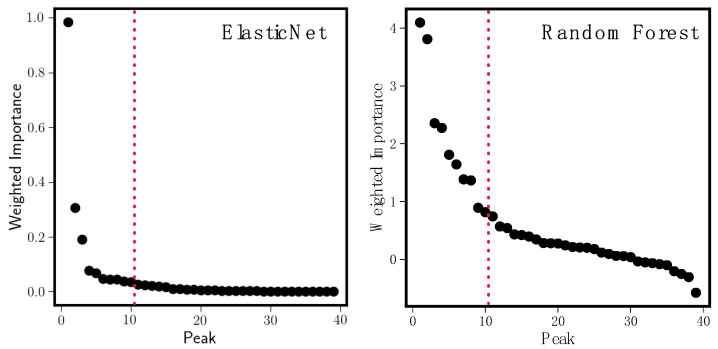
Weighted importance plots for Elastic Net model ensembles (**left**) and Random Forest model ensembles (**right**). Importances have been sorted from largest to smallest and there is no correspondence between peak numbers in the left and right panels. The rapid falloff in importance (extreme in the Elastic Net case) is typical for our method. Dotted lines are drawn after the first ten peaks in each model; these are the peaks we annotate in Table 1.

Figure 4 shows weighted importance plots for model ensembles of size 250 for both the Elastic Net (left panel) and Random Forests (right panel). There is no correspondence between peak order in the left and right panels; each set of weighted importances has been sorted independently in descending order. The steep falloff in importance as we go from left to right in each panel is typical of what we observe in our analyses; of the many available peaks, only a handful are important in predicting bioactivity. An extreme example of this is seen in the Elastic Net model ensemble; the model predicts that, at most, three peaks are important. The Random Forest plot is more typical; while it is difficult to assign a specific cutoff threshold (there is no clear gap between important and unimportant), once we have captured the first eight to ten peaks, we can be confident that we have the meaningful peaks. In what follows, we annotate the first ten peaks in each model ensemble; this is to maintain consistency in comparing findings from the Elastic Net and the Random Forest, despite the fact that the cutoff for the Elastic Net would more naturally fall after the third peak.

**Figure 5:**
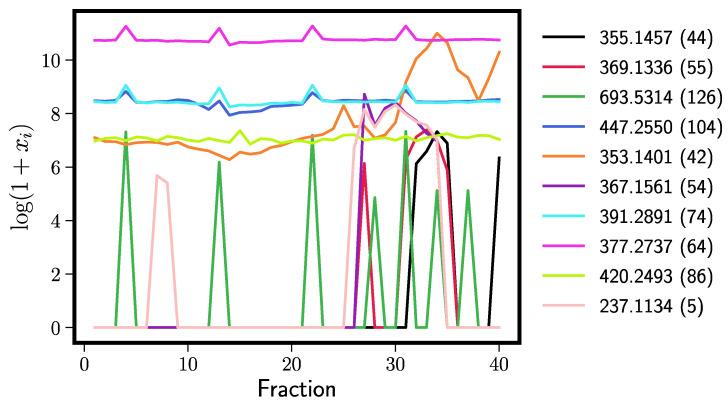
Chromatographic distribution and relative abundance of the top ten Elastic Net predictors of bioactivity across fractions. Color-coded ion currents are indicated by *m*/*z* values and by feature tags in parentheses. See Table 1 for compound names.

**Figure 6:**
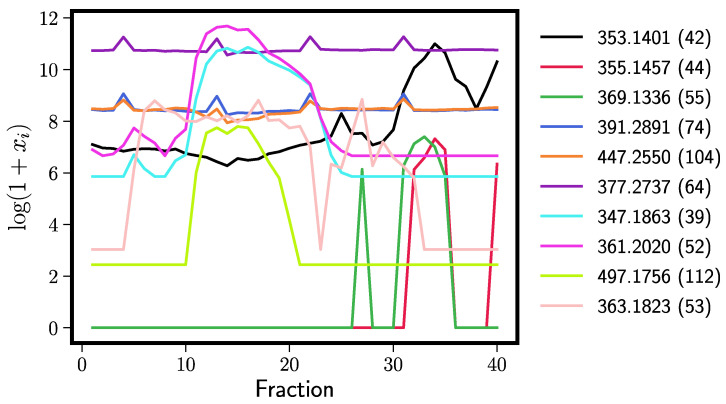
Chromatographic distribution and relative abundance of the top ten Random Forest predictors of bioactivity across fractions. Color-coded ion currents are indicated by *m*/*z* values and feature tags in parentheses. See Table 1 for compound names.

Table 1 shows identifications for the top ten most important Elastic Net peaks (left) and Random Forest peaks (right). Any peak labeled as “noise” is sporadically distributed across the fractions (Figure 5 and Figure 6). These tend to originate from the extract solvent that produces background mass spectra. We see that both models perform well; xanthohumol, which we know from previous research to exert anti-inflammatory effects in vitro [4] and in vivo [5], is the fifth most important hit in the Elastic Net and the most important in Random Forests. Moreover, other xanthohumol derivatives (xanthohumol B, α,β-dihydroxyxanthohumol, 4′-*O*-methylxanthohumol) cluster near the top of the importance list. These structurally related prenylated flavonoids are likely to contribute to the overall iNOS inhibitory effect, but in minor ways, because they occur in the bioactive fractions at orders of magnitude lower concentrations than xanthohumol (see their abundance across fractions in Figure 5 and Figure 6). Random forests also found additional humulones (cohumulone, adhumulone, etc.) that have a predicted anti-inflammatory effect. The humulones are major components of the crude extract (Figure 6), yet they inhibited iNOS activity by no more than 30% compared to the positive control (Figure 1). Therefore, these extract components can be considered weak iNOS inhibitors. The Random Forest models also found fewer noise peaks in the top ten (three vs. five for the Elastic Net).

#### 3.3.2. Global Natural Products Social (GNPS) Molecular Network

**Figure 7:**
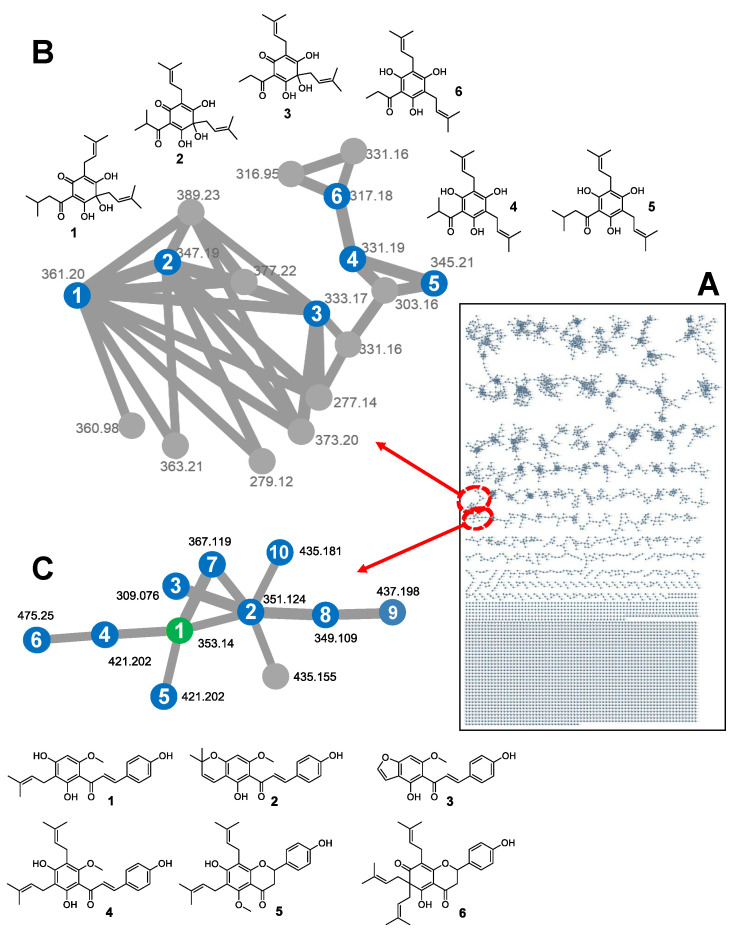
Molecular network generated from the negative ion MS/MS data. (**A**) The network consists of 5122 nodes and 393 clusters, defined as a group of nodes consisting of at least two connected nodes. (**B**) The enlarged humulone cluster shows (ad)humulone **1**, cohumulone **2**, posthumulone **3**, 4-deoxycohumulone **4**, 4-deoxy(ad)humulone **5**, and 4-deoxyposthumulone **6**. (**C**) The enlarged prenylflavonoid cluster shows xanthohumol (**1**, green node) and structurally related prenylated flavonoids (blue nodes): xanthohumol C (**2**, ref. [40]), xanthohumol O (**3**, ref. [44]), 5′-prenylxanthohumol or 5′-prenylisoxanthohumol (**4** or **5**, ref. [39]), isoxantholupon (**6**, ref. [44]), and structurally related but unknown compounds **7**–**10**. All structures are consistent with the accurate masses measured by HR-MS.

In addition to full-scan TOF-MS analysis of the extract fraction, the crude extract was separately analyzed by LC-QTOF-MS/MS analysis to obtain mass fragment (MS/MS) data as input for building a GNPS molecular network, which was visualized in Cytoscape V3.6.1 (Figure 7). MS/MS data were organized in 5122 nodes, which were grouped in 393 clusters. The integration of the Elastic Net approach with GNPS networking resulted in a “primed GNPS” network, predicting that 11 features are related to the observed bioactivity. Using our in-house Oregon Natural Product library of mass spectra (>800 plant secondary metabolites), feature no. 42 was identified as xanthohumol (HRMS [M-H]^−^ *m*/*z* 353.1401 found, calculated for C_21_H_21_O_5_^−^ 353.1394, mass error 2.0 ppm). Integrating GNPS with the computational pipeline described in this study allows us to significantly narrow down a large number of subnetworks GNPS produces to only those that involve model-predicted bioactive compounds (see Figure 7B,C). This could then be used to identify novel compounds in the network by relating them to the identified model compounds; we may be able to identify unknown compounds in the network by linking them to a model compound, as we as show for xanthohumol (green node in Figure 7C). Another example is the tentative identification of 4-deoxyposthumulone (compound **6** in Figure 7B) as a naturally occurring humulone not described in the literature. Compound **6** is structurally related to 4-deoxy(ad)humulone, 4-deoxycohumulone, and posthumulone (compounds **4**–**6** in panel B). Deoxyhumulones are the known biogenetic precursors of (ad)humulone and cohumulone (compounds **1** and **2** in Figure 7B), while it is conceivable that 4-deoxyposthumulone **6** represents the biogenetic precursor of posthumulone **3**.

#### 3.3.3. Validation Study

**Figure 8:**
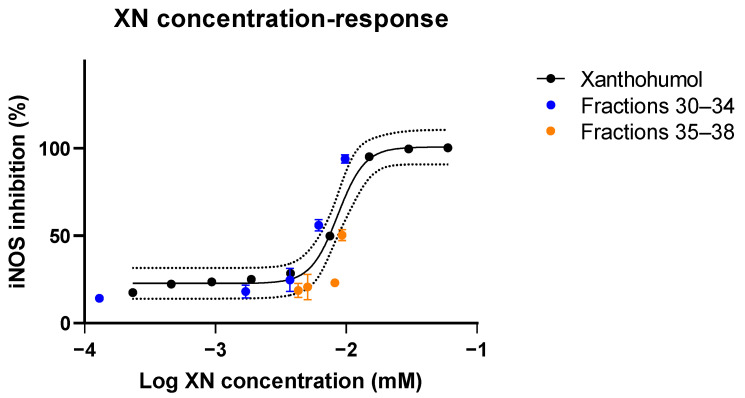
Concentration-response curve of iNOS inhibition by pure xanthohumol (black) and hops extract fractions 30–38. The dotted lines represent 95% prediction bands.

To validate the model’s result, finding xanthohumol as the most important predictor of bioactivity in the iNOS inhibition cell assay, we assayed the dose-response curve for xanthohumol and some of the late chromatographic fractions (30–38; see Figure 8).

Pure xanthohumol-treated cells ranged from a nitrite concentration of 652.1 nmol/mL (0.23 µM XN) to 23.7 nmol/mL (60 µM XN) (Figure 8). The xanthohumol concentrations of the hops fractions 30–38 were determined by quantitative liquid chromatographic and mass spectrometric detection. Concentrations were determined by known preparations of ten-point external calibration curves. The following concentrations of xanthohumol were measured in triplicate for 20 µg/mL hops fractions 30–38: fraction 30, 0.0458 µg/mL (sd ± 0.00113 µg/mL); fraction 31, 0.602 µg/mL (sd ± 0.00323 µg/mL); fraction 32, 1.31 µg/mL (sd ± 0.00639 µg/mL); fraction 332 µg/mL (sd ± 0.00574 µg/mL); fraction 34, 3.46 µg/mL (sd ± 0.0815 µg/mL); fraction 35, 3.29 µg/mL (sd ± 0.0389 µg/mL); fraction 36, 2.91 µg/mL (sd ± 0.0972 µg/mL); fraction 37, 1.80 µg/mL (sd ± 0.0113 µg/mL); and fraction 38, 1.52 µg/mL (sd ± 0.00402 µg/mL) (see Figure 8).

Pure xanthohumol showed a sharp transition to near 100% iNOS inhibition, a curve mirrored by the inhibitory activity of fractions 30–34, in which xanthohumol concentration steadily increased. Fractions 35–38 show low levels of inhibition (a maximum of about 50%) despite significant XN concentrations, indicating that an inhibitor of bioactivity may be present in those fractions. To identify this inhibitor, we conducted the secondary regression analysis described in “Methods,” using the individual peaks as predictors and the difference in predicted (from pure XN) and observed iNOS response in fractions 35–38. The best fitting model (*p* = 0.041, using a one-sided test because we know the direction of effect we expect) was for an *m*/*z* of 293.1781, which is consistent with the compound 2-decyl-4,6-dihydroxybenzoic acid (C_17_H_26_O_4_; mass error −7.4 ppm). This compound is a homologue of olivetolic acid, which is known to be produced in Cannabinaceae [45]. A structurally related compound, anacardic acid, has been reported to stimulate NO production in macrophages [46]. Overall, the validation experiment with pure xanthohumol and our exploration of the antagonistic action of 2-decyl-4,6-dihydroxybenzoate validates our model’s findings regarding xanthohumol.

## 4. Discussion

We have introduced a novel analysis pipeline for the computationally guided discovery of bioactive natural products. Using this method, we obtain robust predictions of bioactive compounds in a problem in which we have many more features (MS peaks) than samples (fractions). Our methodology is flexible and agnostic to the particular statistical learning model employed in the data fitting step; we tested two particular models—the Elastic Net and Random Forests (RF). Others [9,10] have used Partial Least Squares (PLS) to accomplish a similar goal. However, as we have shown here, in extracts in which bioactivity is not broadly shared across many fractions, simply running a single prediction model—without any attention paid to cross-replicate reproducibility filtering or ensemble averaging of multiple models—will yield suboptimal results. Our pipeline solves these issues, and in “easier” cases in which these issues are not a concern, it would produce consistent results with prior, simpler approaches. Moreover, our approach requires less wet-lab work to generate the same or better quality results.

Both the Elastic Net and RF, when inserted into our computational pipeline, predicted xanthohumol to contribute significantly to the hop extract’s anti-inflammatory activity. We validated the findings by testing purified xanthohumol separately in the same iNOS inhibition assay used to assess the chromatographic fractions (Figure 8). Purified xanthohumol showed a strong inhibitory effect, which was mirrored by the inhibitory activity of fractions 30–34 when xanthohumol was in sufficient concentration. One could argue that super-potent xanthohumol analogs could be responsible for the iNOS inhibition. However, we believe this is not the case, as we have previously demonstrated that xanthohumol analogs—including the ones we discussed in the Results section—have roughly equal potency [4]. The bioassay in this previous study was for cytokine production and not iNOS inhibition, but cytokines and iNOS-derived NO are all products of the NFκB pathway. The RF model also found four other humulones which act as weak iNOS inhibitors based on the behavior of the crude extract as compared to the positive control: two of the humulones with predicted bioactivity were very abundant in fractions 10–23 (see Figure 6), but iNOS was inhibited by those fractions by no more than a third of the positive LPS control.

Late fractions (35–38) showed high xanthohumol concentrations but decreasing iNOS inhibition. We performed a secondary analysis on these fractions that identified a candidate antagonist (2-decyl-4,6-dihydroxybenzoic acid) of xanthohumol in those fractions. We note that such interactions can, in principle, be found from the original analysis pipeline if we were to supplement the original model with interaction terms. This comes at the cost of greatly increasing the number of predictor variables; for example, if there are 30 individual peaks to be used as predictors, 435 interaction terms would have to be added to the supplemented model. Verifying the antagonistic effects of 2-decyl-4,6-dihydroxybenzoic acid and employing models with interaction terms are both subjects of future work.

RF performed slightly better than the Elastic Net in that (1) xanthohumol was the top hit in RF, (2) fewer noise peaks were flagged as important in RF, and (3) more xanthohumol derivatives appeared in the set of most important bioactive RF peaks. One could insert PLS (or virtually any other learner) into our pipeline without any difficulty; PLS has natural measures of variable importance [47,48] that would serve in place of the important measures we used for the Elastic Net and RF. However, one benefit to the models we use is that no transformation to latent spaces, which mix together mass peaks, are performed in either the Elastic Net or RF. Our methods directly produce a list of active *m*/*z* peaks, quantified in order of how well they consistently explain bioactivity across all samples, with no further analyses of vector loadings or S-plots.

## 5. Conclusions

We have introduced a flexible, robust machine learning pipeline for natural product discovery and dereplication from mass spectrometry data of complex mixtures. Our models identified xanthohumol as a potent inhibitor of nitric oxide formation in a cell-based assay, which we verified in a subsequent validation statement. Moreover, the models also predicted several other humulones to have inhibitory activity; we were able to identify these molecules as weak inhibitors by comparing the activity of raw extract to a positive control. Our model-based methods require only a single round of fractionation and testing, which could make bioactive compound discovery much more rapid and cost-effective. In our future work, we will determine if including one-dimensional proton NMR measurements along with MS measurements could improve our model-based approach even further.

## Figures and Tables

**Table 1:** Top 10 ensemble bioactive compounds from 250 Elastic Nets (Column 2) and 250 Random Forests (Column 3). Before generating model ensembles, reproducibility filtering was performed using a threshold of r_t_ = 0.71. Feature assignments were made by comparison with in-house library spectra or online database spectra: dihydroxanthohumol (=α,β-dihydroxanthohumol [6]), xanthohumol [39], xanthohumol B [39], 4′-*O*-methylxanthohumol [40], cohumulone [41], adhumulone [42], and cohumulinone [43]. Feature no. 112 was partially characterized as an adhumulone adduct or derivative because it produced fragment ions that were identical to those observed in the MS/MS spectrum of (ad)humulone (feature no. 52). Humulone and adhumulone are isobaric isomers that differ in the substitution of the aromatic ring, i.e., 3-methylbutanoyl versus 2-methylbutanoyl, respectively.

Rank	R_t_ = 0.71, 250 Elastic Nets		R_t_ = 0.71, 250 Random Forests	
	*m*/*z* (Feature Tag)	Feature Assignment	*m*/*z* (Feature Tag)	
1	355.1457 (44)	dihydroxanthohumol	353.1401 (42)	xanthohumol
2	369.1336 (55)	xanthohumol B	355.1457 (44)	dihydroxanthohumol
3	693.5314 (126)	noise	369.1336 (55)	xanthohumol B
4	447.2550 (104)	noise	391.2891 (74)	noise
5	353.1401 (42)	xanthohumol	447.2550 (104)	noise
6	367.1561 (54)	4′-*O*-methyl xanthohumol	377.2737 (64)	noise
7	391.2891 (74)	noise	347.1863 (39)	cohumulone
8	377.2737 (64)	noise	361.2020 (52)	(ad)humulone
9	420.2493 (86)	noise	497.1756 (112)	(ad)humulone adduct/derivative
10	237.1134 (5)	unknown	363.1823 (53)	cohumulinone

## Data Availability

Data and software are available from the authors upon request.
